# Increased plasma renin by vasodilators promotes the progression of abdominal aortic aneurysm

**DOI:** 10.3389/fphar.2023.1174278

**Published:** 2023-06-13

**Authors:** Yu Liu, Shuai Liu, Jiani Zhao, Kemin Wu, Baohui Xu, Wei Wang

**Affiliations:** ^1^ Department of General and Vascular Surgery, Xiangya Hospital, Central South University, Changsha, China; ^2^ National Clinical Research Center for Geriatric Disorders, Xiangya Hospital, Central South University, Changsha, China; ^3^ Department of Surgery, Stanford University School of Medicine, Stanford, CA, United States

**Keywords:** abdominal aortic aneurysm, hypertension, antihypertensive drugs, plasma renin level, plasma renin activity, vasodilator

## Abstract

**Background:** It is well-accepted that antihypertensive therapy is the cornerstone of treatment for abdominal aortic aneurysm (AAA) patients with hypertension. Direct-acting vasodilators were used in the treatment of hypertension by directly relaxing vascular smooth muscle but may have destructive effects on the aortic wall by activating the renin–angiotensin system axis. Their roles in AAA disease remain to be elucidated. In this study, we used hydralazine and minoxidil, two classical direct-acting vasodilators, to investigate their influence and potential mechanisms on AAA disease.

**Methods and results:** In this study, we investigated the plasma renin level and plasma renin activity in AAA patients. Simultaneously, age and gender ratio-matched patients diagnosed with peripheral artery disease and varicose veins were selected as the control group using a ratio of 1:1:1. Our regression analysis suggested both the plasma renin level and plasma renin activity are positively associated with AAA development. In view of the well-established relationship between direct-acting vasodilators and increased plasma renin concentration, we established a porcine pancreatic elastase-infused AAA mouse model, followed by oral administration of hydralazine (250 mg/L) and minoxidil (120 mg/L) to investigate effects of direct-acting vasodilators on AAA disease. Our results suggested both hydralazine and minoxidil promoted the progression of AAA with increased aortic degeneration. Mechanistically, the vasodilators aggravated aortic inflammation by increased leukocyte infiltration and inflammatory cytokine secretion.

**Conclusion and relevance:** The plasma renin level and plasma renin activity are positively associated with AAA development. Direct vasodilators aggravated experimental AAA progression, which raised cautionary concerns about their applications in AAA disease.

## Introduction

Abdominal aortic aneurysm (AAA) is the dilation of the abdominal aorta 1.5 times its original size, generally more than 3 cm, with structural disruptions of the aortic vasculature (Golledge, 2019). The incidence of AAAs increases significantly in men aged >55 years and women aged >70 years, posing a high risk to the elderly due to the high morbidity and mortality associated with aortic rupture (Bossone and Eagle, 2021). Although previous studies reported successful medical stabilization of growing AAAs in different animal models, the current management of AAA mainly relies on the prophylactic operative repair of larger aneurysms (Liao et al., 2001; Golledge et al., 2006; Sakalihasan et al., 2018). Currently, medical management during aneurysm surveillance warrants further studies.

Arterial hypertension is an important population-attributable risk factor for AAA, which is linked to an increased risk of cardiovascular events and adverse prognosis, following AAA (Kobeissi et al., 2019). Previous studies reported that for every 20-mmHg increase in systolic blood pressure and 10-mmHg increase in diastolic blood pressure, the relative risk for AAA increases by 14% and 28%, respectively (Kobeissi et al., 2019; Wanhainen et al., 2019). Although antihypertensive therapy has not been shown to inhibit aneurysm expansion, it could benefit patients by reducing the risks of cardiovascular events (Bicknell et al., 2016). Thus, guidelines encouraged AAA patients to seek appropriate medical management for hypertension (Chaikof et al., 2018; NICE guideline [NG156]). However, specific recommendations for the choice of antihypertensive medication remain poorly described, which is mainly referred to the local guidelines, as demonstrated in the ESVS and NICE guidelines (Wanhainen et al., 2019).

Direct-acting vasodilators are a heterogeneous group of drugs, which can directly dilate peripheral arterioles and lower blood pressure, and are generally used in systemic hypertension, especially in patients with refractory hypertension, stage III hypertension, or renal dysfunction (Cassis et al., 2009; Lu et al., 2012). Despite the decline in their use due to adverse effects, direct vasodilators are still valuable in clinical practice for these niche indications. Hydralazine and minoxidil are the most commonly prescribed direct vasodilators for hypertension (Laurent, 2017). However, it is reported that arterial vasodilation induced by direct vasodilators can activate the peripheral sympathetic nervous system via carotid and aortic baroreceptor reflexes, casing increased circulating renin levels and subsequent activation of the renin–angiotensin system, which eventually leads to tachycardia and fluid retention (Gottlieb et al., 1972; Campese et al., 1979; Sica and Gehr, 2001). As the only enzyme known to cleave angiotensinogen and the rate-limiting enzyme of the renin-angiotensin system, plasma renin can increase the synthesis of angiotensin II and aldosterone, which plays a critical role in cardiovascular disease (Ferrario and Strawn, 2006; Miyake et al., 2017). Considering the essential role of the renin–angiotensin system in the progression of AAA disease, it is worth investigating whether neurohumoral regulation by direct-acting vasodilators aggravates AAA disease.

In the study, we investigated the relationship between the plasma renin level (PRL) and plasma renin activity (PRA) with AAA in patients using a case–control design, which suggested PRL and PRA are positively associated with AAA development. Given that direct vasodilators may increase the PRL and PRA, we explored the effect of direct vasodilators on AAA disease using hydralazine and minoxidil. Our results suggested direct vasodilators aggravated experimental AAA progression, raising cautionary concerns about their applications on arterial disease and AAA disease.

## Methods

### Study subjects

#### Cases

Patients diagnosed with AAA were selected for pre-onset PRL and PRA determination according to the disease diagnosis code of the 10th edition of the International Classification of Diseases (ICD) as the case group. AAA is defined as 50% dilation of the abdominal aorta, generally more than 3 cm of normal (Golledge, 2019). At the same time, two investigators examined and determined the aortic angiography and computed tomography findings and classification of all cases to exclude false positives. Patients with connective tissue disease, Marfan syndrome, vasculitis, and traumatic aortic dissection were excluded. In addition, patients who had a definite diagnosis of secondary hypertension at the time of aldosterone testing (renal vascular hypertension, renal hypertension, and aldosterone-producing adenoma with adenoma larger than 1 cm, Cushing’s syndrome, pheochromocytoma, and coarctation of the aorta) were excluded.

#### Control

Given the possibility of increased plasma renin levels in arterial disease (Marchesi et al., 2008), we included peripheral arterial disease (PAD) and varicose vein (VV) as controls using a case–control ratio of 1:1:1 matching age and sex with the same exclusion criteria. In addition, in order to explore the effects of AAA on the PRL and PRA, the PAD and VV groups were combined into the non-AAA group and compared with the AAA group.

#### Data collection

We obtained patient data from the hospital electronic medical database. The following general, clinical, and laboratory data were collected: general data (age, gender, and cigarette consumption), anthropometric data [body mass index, systolic blood pressure, diastolic blood pressure, pulse rate, and abdominal aorta diameter], biochemical measurements [total cholesterol, triglycerides, LDL cholesterol, HDL cholesterol, alanine aminotransferase, aspartate aminotransferase, serum creatinine, serum uric acid, serum potassium, serum sodium, plasma renin level (PRL), and plasma renin activity (PRA)], comorbidities [coronary heart disease diabetes, hypertension, chronic obstructive pulmonary diseases, and chronic kidney disease], and medication history (hypoglycemic medicine, lipid-lowering drugs, and antihypertensive drugs).

#### PRL and PRA measurements

After approval of Xiangya Hospital Institutes’ Review Board and Ethics Committee of the Xiangya Hospital, Central South University, blood collection was carried out at dawn of the second day of patients’ admission to ensure the measurements of recumbent position PRL and PRA are comparable. Then, the blood samples were transported to and evaluated at the clinical laboratory of Xiangya Hospital immediately.

#### Animals and animal care

Ten-week-old male C57BL/6J mice were purchased from the Xiangya School of Medicine, Central South University. Mice were given unique numbers after quarantine and then randomly divided into a vehicle group, hydralazine group, and minoxidil group, according to different administrations by stratified random sampling. The mice were housed under specific pathogen-free conditions in a 12-h light/dark cycle with *ad libitum* access to food and water. Calculation of the sample size was determined by power analysis, as previously reported (Festing and Altman, 2002). According to previous research on experimental AAA disease, *n* = 8–15 specimens per group are required. The sample size was calculated by referring to the previously published literature of our research group, and a total of 40 mice were used in this study (Liu et al., 2020). Each mouse constituted an experimental unit. The mice were divided into the vehicle group (*n* = 15), hydralazine group (*n* = 9), and minoxidil group (*n* = 13), and we declared no excluding animals. Hydralazine (250 mg/L) and minoxidil (120 mg/L) were administered via drinking water and were replaced every other day. The dosage, time points, and route of administration were chosen based on the prior literature (Tsoporis et al., 1998; Knutsen et al., 2018; Kobeissi et al., 2019).

#### Porcine pancreatic elastase (PPE)-induced AAA model in mice

AAAs were created via transient intra-infrarenal aortic infusion of PPE, based on the study conducted by Liu et al. (2022). Mice were randomly selected from each cage in turn, according to the stratified sampling results for the following steps. Briefly, the mice were anesthetized with inhaled isoflurane, and surgical procedures were performed under sterile conditions. The mouse was subjected to transient infusion of type I PPE (4.0 U/mL; E1250; Sigma-Aldrich) dissolved in saline at a pressure of 150 mmHg. The aortic diameters of pre-infusion and post-infusion were measured to ensure consistency and minimize potential confounders for each group (Liu et al., 2022). Following the closure of laparotomy, all mice were housed under specific pathogen-free conditions as the preoperative environment. At the end, mice were anesthetized using ketamine/xylazine (100 and 20 mg/kg i.p., respectively) and euthanized by exsanguination before aortic tissue collection on day 14. AAA was defined as more than 50% enlargement of the maximum abdominal aortic diameter. All processes were implemented under double‐blind conditions.

#### Imaging AAA formation and progression

AAA was monitored via serial transabdominal high-frequency ultrasound (Vevo^®^ 2100 Imaging System, VisualSonics, Toronto, ON, Canada) for determining the internal diameter or using a digital camera for the external diameter. Measurements of the aortic extra-diameter were performed before and after perfusion on day 0, and before euthanasia on day 14. Measurements of the aortic intro-diameter were performed on days 0 and 14 by investigators blinded to the group assignment.

#### Histological analyses

Mice were euthanized 14 days after PPE infusion. Aortae were harvested, fixed in 4% paraformaldehyde, embedded in paraffin, and horizontally cut into sections. For histological analyses, hematoxylin–eosin (H&E) staining, Masson, elastic Van Gieson (EVG) staining, and a two-step standard immunoperoxidase procedure for immunohistochemistry were conducted to identify CD3^+^ T cells (ab16669; Abcam), CD68^+^ macrophages (ab125212; Abcam), CD31^+^ mural vessels (ab28364; Abcam), and MMP9 (ab38898; Abcam). The same concentration of rabbit control IgG (AC005, ABclonal) serves as the negative control.

#### Real-time polymerase chain reaction (PCR)

Mice were euthanized 14 days after PPE infusion. The total RNA of aortae was extracted using the TRIzol reagent (TaKaRa, Japan), according to the manufacturer’s instruction. A measure of 500 nanograms of total RNA was used for reverse transcription using a PrimeScript RT Reagent Kit (TaKaRa). Quantitative RT-PCR on a real-time PCR system (Applied Biosystems) was performed using SYBR Premix Ex Taq qRT-PCR assays (TaKaRa). The Ct value of mRNA was normalized to GAPDH, and the fold change was calculated using the ΔΔCt method. The primer sequences for genes are listed as follows: ACTB, forward primer: *GTG​CTA​TGT​TGC​TCT​AGA​CTT​CG* and reverse primer: *ATG​CCA​CAG​GAT​TCC​ATA​CC*; NOS2, forward primer: *GTT​CTC​AGC​CCA​ACA​ATA​CAA​GA* and reverse primer: *GTG​GAC​GGG​TCG​ATG​TCA​C*; CCL2, forward primer: *TAA​AAA​CCT​GGA​TCG​GAA​CCA​AA* and reverse primer: *GCA​TTA​GCT​TCA​CAT​TTA​CGG​GT*; IFN-γ, forward primer: *CAG​CAA​CAG​CAA​GGC​GAA​AAA​GG* and reverse primer: *TTT​CCG​CTT​CCT​GAG​GCT​GGA​T*; IL-1β, forward primer: *GAA​ATG​CCA​CCT​TTT​GAC​AGT​G* and reverse primer: *TGG​ATG​CTC​TCA​TCA​GGA​CAG*.

#### Statistical analysis

Continuous variables are expressed as mean values ± standard deviation, unless otherwise stated. For the duration of hypertension, triglyceride, alanine aminotransferase, aspartate aminotransferase, serum creatinine, serum uric acid, PRL, and PRA, the medians in the 25th and 75th percentile ranges are provided because they do not satisfy the normal distribution. One- or Two-way ANOVA was used to compare the normal distribution of continuous variables including age, body mass index, systolic blood pressure, diastolic blood pressure, pulse rate, AAA diameter (for both humans and mice), low-density lipoprotein cholesterol, high-density lipoprotein cholesterol, serum K^+^, and serum Na^+^ among the three groups, and the independent sample *t*-test was used between two groups. If the data did not meet the normal distribution, the quantile-quantile graph was used to check its distribution. If the data were located near the diagonal, there was an approximate normal distribution between the groups (including total cholesterol and low-density lipoprotein cholesterol). Non-parametric tests (including Kruskal Wallis and Mann Whitney tests) were provided to compare the difference among ranked data and continuous variables that do not satisfy normal distribution or homogeneity of variance (including the duration of hypertension, triglyceride, ALT, AST, serum creatinine, serum uric acid, PRL, PRA, EVG, and SMA score). We used the χ2 analysis (χ2 test) to compare categorical variables, frequencies, and proportions (including sex, cigarette consumption, coronary heart disease, diabetes, hypertension chronic obstructive, pulmonary diseases, chronic kidney disease, and medication history). Multivariate logistic regression analysis was used to evaluate the relationship between the PRL, PRA and AAA among the three groups, and binary logistic regression analysis was used for two groups. The multivariate linear regression analysis was used to test the collinearity between variables. Odds ratio and 95% CI were reported. A two-tailed *p*-value of 0.05 is considered significant. SPSS 26.0 version was used for data management and analysis.

## Result

### Baseline characteristics of patients enrolled

Finally, 20 patients diagnosed with AAA from 01/01/2022 to 01/11/2022 were enrolled in the case group. Meanwhile, 20 patients diagnosed with PAD and VV matched with age and sex were included, respectively. The baseline characteristics of the 60 patients are given in Table 1. As is shown, cigarette consumption, coronary heart disease, the diabetes composition ratio, and systolic blood pressure revealed significant differences among the three groups (Table 1). Specifically, the diabetes composition ratio (10% versus 50%, AAA versus PAD, and *p* = 0.014) showed statistically significant differences between AAA and other two groups when compared in a pairwise manner (Table 1). Statistical differences in cigarette consumption (75% versus 35%, PAD versus VV, and *p* = 0.031), the coronary heart disease composition ratio (60% versus 20%, PAD versus VV, and *p* = 0.035), and systolic blood pressure (144.5 ± 17.58 mmHg versus 129.1 ± 15.41 mmHg, PAD versus VV, and *p* = 0.026) were observed when comparing the PAD group with the VV group (Table 1).

**TABLE 1:** Baseline characteristics of the patients enrolled.

	VV (*n* = 20)	PAD (*n* = 20)	AAA (*n* = 20)	*p*-value	*p*-value with pairwise comparisons		
					AAA vs. VV	AAA vs. PAD	PAD vs. VV
Age (years)	67 ± 6.02	68.9 ± 6.83	67.6 ± 6.34	0.633			
Gender (women/men), *n* (%)	3/17 (15/85)	3/17 (15/85)	3/17 (15/85)	1.000			
BMI	24.75 ± 2.5	22.2 ± 3.19	22.99 ± 3.74	0.086			
Cigarette consumption (yes), *n* (%)	7 (35)	15 (75)	14 (70)	0.029	0.075	1.000	0.031
CAD, *n* (%)	4 (20)	12 (60)	11 (55)	0.025	0.082	1.000	0.035
Diabetes, *n* (%)	4 (20)	10 (50)	2 (10)	0.018	1.000	0.014	0.100
Hypertension, *n* (%)	12 (60)	17 (85)	14 (70)	0.250			
COPD, *n* (%)	0	4 (20)	5 (25)	0.062			
CKD, *n* (%)	2 (10)	4 (20)	4 (20)	0.749			
Duration of hypertension, y	0 (0∼8.25)	9.00 (0∼10.00)	5.00 (0∼10.00)	0.104			
Systolic blood pressure, mm Hg	129.1 ± 15.41	144.5 ± 17.58	136.55 ± 17.51	0.020	0.503	0.425	0.026
Diastolic blood pressure, mm Hg	80.9 ± 10.11	83.6 ± 12.21	78.35 ± 15.89	0.445			
Pulse rate, beats per minute	75.45 ± 14.06	75.4 ± 10.92	75.45 ± 14.69	1.000			
Total cholesterol, mmol/L	4.75 ± 1.08	4.42 ± 0.93	4.83 ± 2.14	0.681			
Triglyceride, mmol/L	1.34 (1.02∼2.42)	1.74 (1.03∼2.40)	1.59 (0.98∼1.99)	0.995			
LDL-C, mmol/L	3.07 ± 0.75	2.83 ± 0.74	3.03 ± 1.1	0.667			
HDL-C, mmol/L	1.1 ± 0.29	1.02 ± 0.26	1.05 ± 0.26	0.661			
ALT, U/L	18.40 (13.03∼22.13)	15.50 (12.24∼23.13)	14.65 (9.93∼18.98)	0.314			
AST, U/L	22.10 (17.45∼29.55)	20.25 (16.25∼25.53)	20.20 (17.85∼25.40)	0.557			
Serum creatinine, μmol/L	70.25 (58.75∼82.75)	83.25 (69.00∼93.20)	89.60 (67.95∼103.08)	0.191			
Serum uric acid, μmol/L	367.75 (311.03∼496.12)	344.40 (333.63∼447.80)	437.15 (306.63∼514.38)	0.627			
Serum potassium, mmol/L	4.04 ± 0.53	3.92 ± 0.4	3.97 ± 0.4	0.693			
Serum sodium, mmol/L	141.62 ± 1.87	140.86 ± 3.61	141.37 ± 3.4	0.728			
Abdominal aorta diameter, mm		17.89 ± 1.68	59.72 ± 17.33			<0.001	
Hypoglycemic medicine, *n* (%)	2 (10)	5 (25)	1 (5)	0.246			
Lipid-lowering drugs, *n* (%)	3 (15)	6 (30)	5 (25)	0.641			
Antihypertensive drugs							
CCB, *n* (%)	8 (40)	12 (60)	9 (45)	0.521			
ACEI/ARB, *n* (%)	4 (20)	2 (10)	6 (30)	0.416			
β-blocker, *n* (%)	4 (20)	3 (15)	6 (30)	0.630			
Diuretic, *n* (%)	2 (10)	3 (15)	2 (10)	1.000			

Note: Values represent means ± standard deviation or count and percentage where otherwise specified. Data satisfying normal distribution and homogeneity of variance were analyzed by single-factor ANOVA; if not, the non-parametric (K-W) test was applied. Pairwise comparison was applied for data with *p* < 0.05 in the multigroup comparison. The independent sample *t*-test was used for abdominal aorta diameter data (PAD vs. AAA). VV, varicose veins; PAD, peripheral artery disease; AAA, abdominal aortic aneurysm; BMI, body mass index; CAD, coronary artery disease; COPD, chronic obstructive pulmonary disease; CKD, chronic kidney disease; LDL-C, low-density lipoprotein cholesterol; HDL-C, high-density lipoprotein cholesterol; ALT, alanine aminotransferase; AST, aspartate aminotransferase; CCB, calcium channel blocker; ACEI, angiotensin-converting enzyme inhibitor; ARB, angiotensin receptor blocker.

Considering the critical role of renin in vascular disease (Wu et al., 2018), the PAD and VV groups were roughly combined into the non-AAA group to exclude the bias of the plasma renin level and activity in arterial disease. Characteristics of AAA and non-AAA groups are given in Supplementary Table S1. The AAA group showed no significant differences compared to the non-AAA group.

### PRL and PRA are increased in AAA disease

**FIGURE 1:**
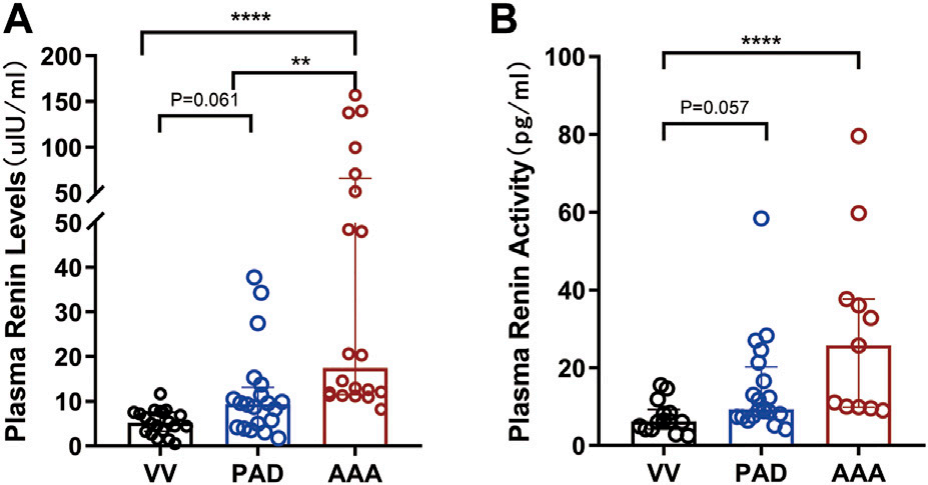
Comparison of PRL and PRA among AAA, PAD, and VV groups. Plasma renin levels and renin activity were measured in the included patients to evaluate the differences in plasma renin levels and activity among the AAA (*n* = 20), PAD (n = 20), and VV (*n* = 20) groups. **(A)** Plasma renin level (PRL) measurement (left). Data were expressed as the median with an interquartile range. **(B)** Plasma renin activity (PRA) measurement (right). Data were expressed as the median with an interquartile range. The K-W test and pairwise comparisons were applied. **p < 0.01 and ****p < 0.0001.

As shown in Table 2 and Figure 1, the AAA group showed a significantly higher PRL [17.47 (11.48∼65.86) versus 9.42 (4.35∼13.21) versus 5.24 (3.28∼7.55), AAA versus PAD versus VV ulU/ml, and *p* < 0.001] and higher PRA [25.72 (9.89∼37.64) versus 9.29 (8.42∼20.16) versus 6.17 (4.21∼9.24), AAA versus PAD versus VV pg/ml, and *p* < 0.001] than the PAD and VV groups. Similarly, compared to the non-AAA group, the AAA group also showed a higher PRL [17.47 (11.48∼65.86) versus 7.02 (3.67∼9.65) ulU/ml, and *p* < 0.001] and higher PRA [25.72 (9.89∼37.64) versus 8.28 (6.05∼13.45) pg/ml, and *p* = 0.001] (Supplementary Table S2; Supplementary Figure S1).

**TABLE 2:** Comparison of PRL and PRA among AAA, PAD, and VV groups.

	Groups			H-value (K-W test)	*p*-value
	VV (*n* = 20)	PAD (*n* = 20)	AAA (*n* = 20)		
PRL, μlU/ml	5.24 (3.28∼7.55)	9.42 (4.35∼13.21)	17.47 (11.48∼65.86)	31.83	<0.001
PRA pg/ml	6.17 (4.21∼9.24)	9.29 (8.42∼20.16)	25.72 (9.89∼37.64)	15.39	<0.001

Note: PRL, plasma renin level; PRA, plasma renin activity.

### PRL and PRA are positively associated with AAA development

Multiple logistic regression analysis was conducted to identify whether the progression of abdominal aortic aneurysms could be attributed to increased plasma renin levels and activity. It is important to mention that a detailed collinearity analysis (SPSS collinearity tests) was performed between the PRL and PRA, with the piece of evidence that the Spearman correlation coefficient of the PRL and PRA is 0.883 (Supplementary Table S3). Therefore, we analyzed the two indicators separately. A univariate analysis was conducted to evaluate the relationship between the increased PRL, and PRA and AAA development. Our results showed higher PRL was significantly associated with AAA development with an unadjusted OR of 1.456 [(95% CI, 1.180–1.797), and *p* < 0.001] compared with VV and 1.064 [(95% CI, 1.002–1.130), and *p* = 0.041] compared with PAD (Table 3). Similarly, higher PRA was also associated with AAA development with an unadjusted OR of 1.280 [(95% CI, 1.045–1.568), and *p* = 0.017] compared with VV (Table 3). Similar trends were observed after adjustment of hypertension and the chronic kidney disease composition ratio in model 1, although the difference was not significant in model 2 (Table 3). Surprisingly, we also found that increased PRL and PRA were correlated with a higher risk of PAD (unadjusted OR, 1.368 and 1.219, respectively) than the VV group (Table 3).

**TABLE 3:** Multiple logistic regression analyses for the association between PRL/PRA and AAA.

				Unadjusted model		Model 1		Model 2	
				OR (95% CI)	*p*-value	OR (95% CI)	*p*-value	OR (95% CI)	*p*-value
PRL, μlU/ml		AAA vs. VV		1.456 (1.180–1.797)	<0.001	1.665 (1.258–2.204)	<0.001	1.667 (1.256–2.213)	<0.001
		PAD vs. VV		1.368 (1.114–1.681)	0.003	1.561 (1.183–2.059)	0.002	1.574 (1.190–2.083)	0.001
		AAA vs. PAD		1.064 (1.002–1.130)	0.041	1.067 (1.006–1.132)	0.032	1.059 (0.995–1.127)	0.069
PRA, pg/ml		AAA vs. VV		1.280 (1.045–1.568)	0.017	1.359 (1.064–1.735)	0.014	1.388 (1.068–1.804)	0.014
		PAD vs. VV		1.219 (1.000–1.487)	0.050	1.286 (1.011–1.637)	0.040	1.303 (1.006–1.686)	0.045
		AAA vs. PAD		1.050 (0.998–1.105)	0.061	1.056 (1.000–1.115)	0.050	1.066 (0.999–1.137)	0.054

Note: Model 1 was adjusted for the hypertension and chronic kidney disease composition ratio. Model 2 was adjusted for the hypertension, chronic kidney disease and diabetes composition ratio.

In addition, a binary logistic regression was conducted between AAA and non-AAA groups, which demonstrated that both high-level PRL and PRA were positively associated with AAA development with an unadjusted OR of 1.099 [(95% CI, 1.026–1.176), and *p* = 0.007] for the PRL and 1.070 [(95% CI, 1.013–1.129), and *p* = 0.014] for PRA, and adjusted OR of 1.100 [(1.028–1.176); *p* = 0.005] for PRL and 1.075 [(1.015–1.138); *p* = 0.014] for PRA in model 1 and 1.102 [(1.024–1.186); *p* = 0.009] for PRL and 1.088 [(1.019–1.162); *p* = 0.012] for PRA in model 2, respectively (Supplementary Table S4).

As PAD and AAA shared similar etiology and pathological mechanisms, the elevated risk of AAA was reported in PAD patients (Hicks et al., 2021; Cai et al., 2022). Thus, a correlation analysis was performed to determine the relationship between aortic diameters and PRL and PRA in AAA and PAD groups. Our results demonstrated a significant positive correlation between the aortic diameter and both PRL (*γ* = 0.529; *p* < 0.001) and PRA (*γ* = 0.412; *p* = 0.021) (Supplementary Table S5). However, the correlation was not significant in the AAA group (*γ* = −0.025; *p* = 0.917) and PRA (*γ* = −0.335; *p* = 0.285).

### Influence of direct-acting vasodilators on PRL and PRA

Hydralazine and minoxidil, the most commonly prescribed direct-acting vasodilators in hypertension (Laurent, 2017), were selected as representatives of direct vasodilators in our study. Given the well-established relationship between the administration of direct-acting vasodilators and PRL and PRA, a review of the published literature showed that the administration of minoxidil and hydralazine increased the PRL and PRA in both clinical and animal experiments, as shown in Table 4.

**TABLE 4:** Influence of hydralazine and minoxidil on PRL and PRA in published studies.

Vasodilator	Species	Dose and route	Effect on PRL and PRA	Effect on blood pressure	Reference
Hydralazine	Human	Varies with individuals, Q6h, oral administration	Elevated PRL compared with premedication (14.5 vs. 35.9 mug/ml/hr)	Lower blood pressure than premedication (191/128 vs. 169/108 mmHg; *p* < 0.01)	Gottlieb et al. (1972)
	Human	150 mg, Qd, oral administration	Elevated PRL compared with premedication (19 ± 3 vs. 25 ± 4 mIU/L; *p* = 0.067)	Lower blood pressure than premedication, with the aim of 140/90 mmHg	Veldhuizen et al. (2021)
	Human	Not available	Elevated PRA	Lower arterial blood pressure than premedication (107.0 ± 2.0 vs. 124.2 ± 3.7 mmHg; *p* < 0.01)	Velasco and McNay (1977)
	Rat	Concentration gradient administration, 0.1, 0.3, 1.0, and 6.0 mg/kg, intraperitoneal injection	PRA increased with dose and peaked at 20 min	Not available	Pettinger et al. (1973)
	Mouse	15 mg/kg, infused by osmotic minipumps subcutaneously	Increased mouse kidney renin mRNA 2.4-fold (248% ± 62% of vehicle)	Lower arterial blood pressure than premedication (73 ± 1 vs. 100 ± 2 mmHg)	Keen and Sigmund (2001)
Minoxidil	Human	Varies with individuals, Q6h, oral administration	Elevated PRL, but lower than hydralazine administration (31.1 vs. 35.9 mug/ml/hr)	Minoxidil was more effective in lowering blood pressure than hydralazine (169/108 vs. 142/92 mmHg; *p* < 0.05)	Gottlieb et al. (1972)
	Human	Start with 5 mg, doubling the dose every 6 h, oral administration	Elevated PRA compared with premedication (8.58 ± 2.83 vs. 1.12 ± 0.28 ng/ml/hr; *p* < 0.02)	Lower mean arterial blood pressure than in the control group (110.6 ± 3.1 vs. 141.2 ± 4.3 mmHg; *p* < 0.001)	Velasco et al. (1974)
	Human	Start with 5 mg, and doubling the dose every 6 h, up to a maximum of 20 mg at a single dose, oral administration	Elevated PRA compared with premedication (7.29 ± 2.68 vs. 1.03 ± 0.26 ng/ml/hr; *p* < 0.05)	Lower mean arterial blood pressure than that of the control group (108.5 ± 3.0 vs. 140.1 ± 5.1 mmHg; *p* < 0.001)	O'Malley et al. (1975)
	Rat	Concentration gradient administration, 0.3, 1.0, 6.0, and 30.0 mg/kg, intraperitoneal injection	PRA increased with dose and peaked at 45 min	Not available	Pettinger et al. (1973)

### Direct-acting vasodilators aggravated experimental AAA

**FIGURE 2:**
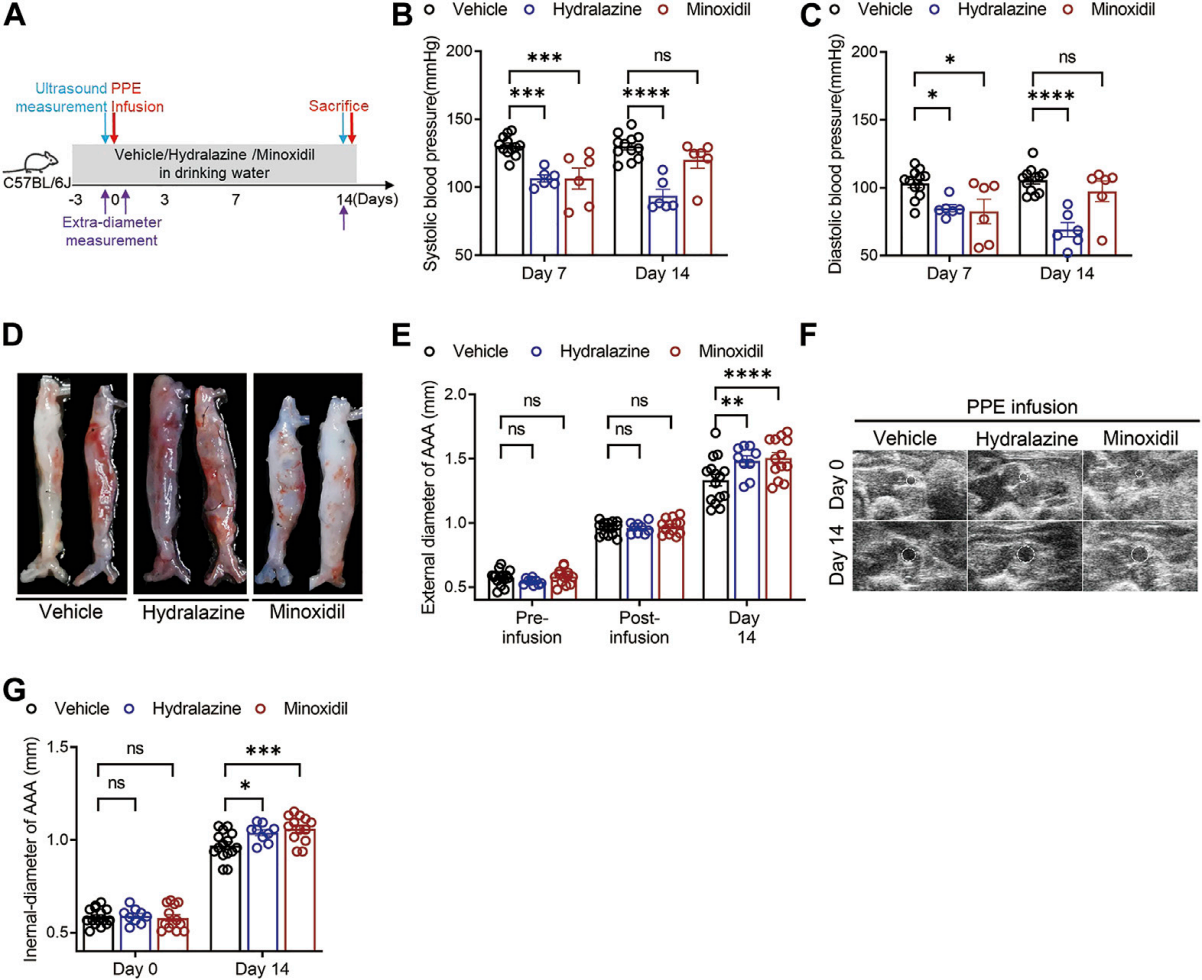
Hydrazine and minoxidil aggravated experimental AAA disease. **(A)** Scheme of the PPE-induced experimental AAA mouse model and monitoring of AAA diameters. **(B,C)** Effect of hydrazine and minoxidil on systolic blood pressure and diastolic blood pressure in experimental AAA. **(D,E)** Representative AAA images and quantitative analysis of aortic extra-diameters in the vehicle group (*n* = 15), hydrazine group (*n* = 9), and minoxidil group (*n* = 13). **(F,G)** Representative ultrasound AAA images and quantitative analysis of aortic intra-diameters and growth rate in the three groups. Data are presented as the mean ± SEM. Significance was analyzed using two-way ANOVA with the Bonferroni correction. *p < 0.05; **p < 0.01; and ***p < 0.001 vs. vehicle group.

Considering the critical role of renin in AAA disease, we then established a PPE-induced experimental AAA mouse model to verify whether direct-acting vasodilators aggravated AAA development and progression. Hydrazine, minoxidil, and vehicle treatment were administrated 3 days before PPE infusion until euthanasia, while blood pressure and aortic diameters were monitored (Figure 2A). Our results suggested hydralazine administration significantly decreased systolic blood pressure and diastolic blood pressure compared to the vehicle group on days 7 and 14, respectively (Figure 2B). Although minoxidil-treated mice displayed a significant decrease in blood pressure (106.3 ± 19.04 versus 130.41 ± 7.18 mmHg and *p* < 0.001 for systolic blood pressure 82.45 ± 22.17 versus 103.06 ± 10.45 mmHg and *p* = 0.007 for diastolic blood pressure) on day 7, this significant difference disappeared on day 14 (Figures 2B, C), supporting the notion that the neurohumoral changes induced by minoxidil can offset its benefits to arteriolar vasodilatation (Baer et al., 1980; Sica and Gehr, 2001).

Monitoring external diameters using a digital camera, our results suggested AAA diameters in the three groups were comparable after PPE infusion but increased more rapidly in hydrazine and minoxidil groups than those in the vehicle group (1.48 ± 0.12 mm versus 1.33 ± 0.17 mm, hydralazine versus vehicle, *p* = 0.037; 1.50 ± 0.15 mm versus 1.33 ± 0.17 mm, minoxidil versus vehicle, *p* = 0.018) (Figures 2D, E). These images of all aneurysms are given in Supplementary Figure S2A. Likewise, hydrazine and minoxidil aggravated AAA progression, which was observed by monitoring internal diameters by ultrasonography (1.04 ± 0.05 mm versus 0.97 ± 0.07 mm, hydralazine versus vehicle, and *p* = 0.017; 1.06 ± 0.07 mm versus 0.97 ± 0.07 mm, minoxidil versus vehicle, and *p* < 0.001).

### Direct-acting vasodilators promoted aortic degeneration

**FIGURE 3:**
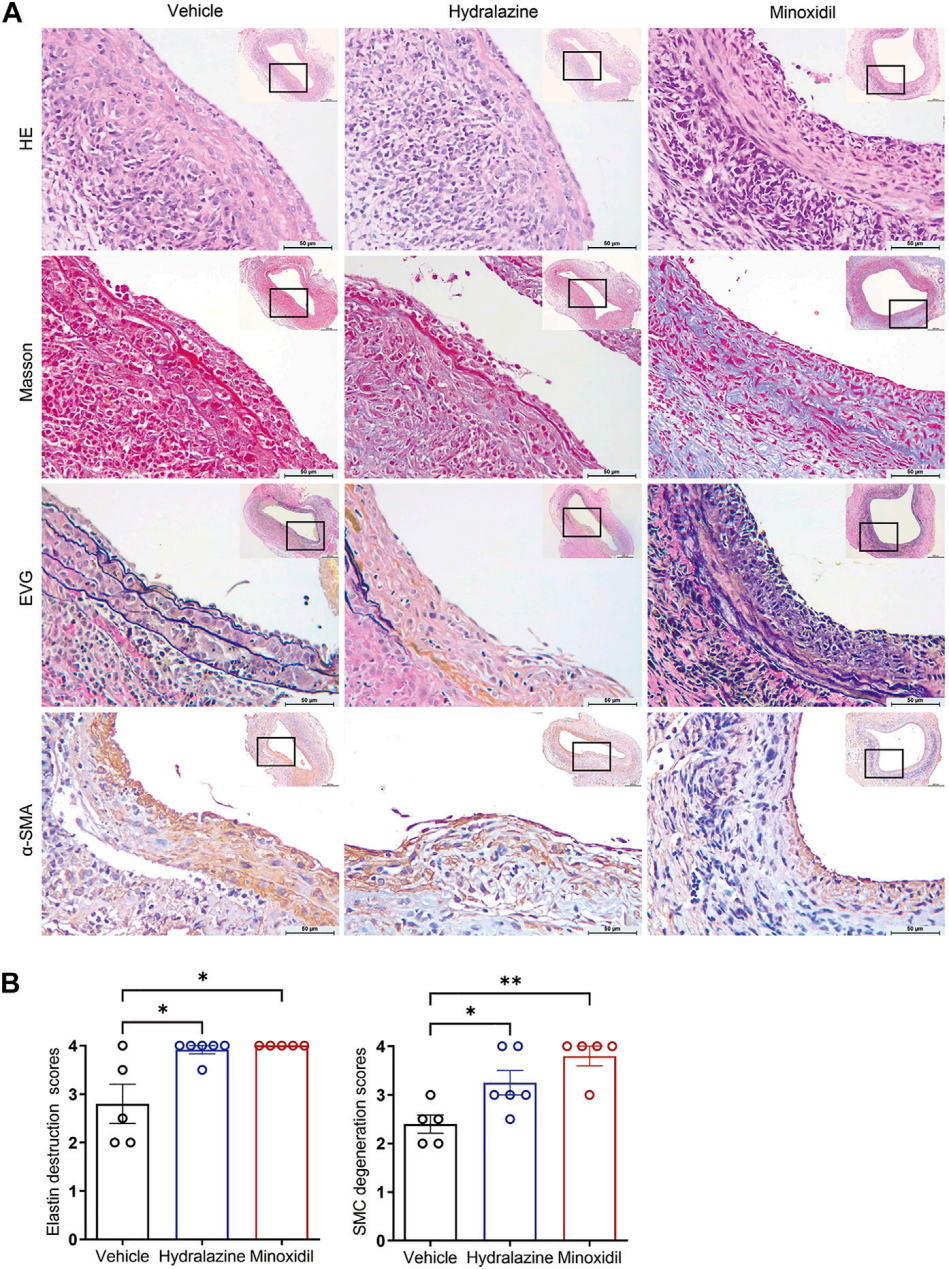
Hydrazine and minoxidil promoted aortic degeneration in experimental AAA. **(A)** Representative images of HE, Masson, EVG, and anti-SMC α-actin staining in the vehicle group (*n* = 5), hydrazine group (*n* = 6), and minoxidil group (*n* = 5). **(B)** Quantitative analysis of EVG and anti-SMC α-actin staining in the three groups. Data are presented as the mean ± SEM. Significance was analyzed using the Mann–Whitney test. *p < 0.05; **p < 0.01 vs. vehicle group.

Next, histological analyses demonstrated that both hydrazine and minoxidil promoted inflammatory cell infiltration and deposited collagen fibers compared to the vehicle group in H&E and Masson staining (Figure 3A). Furthermore, hydrazine and minoxidil aggravated vascular structure disorder, as evidenced by increased elastic destruction scores and vascular smooth muscle cell degeneration scores qualified by EVG and α-SMA staining (Figures 3A, B). These results histologically confirmed aortic degeneration was aggravated by hydrazine/minoxidil administration.

### Direct-acting vasodilators aggravated aortic inflammation

**FIGURE 4:**
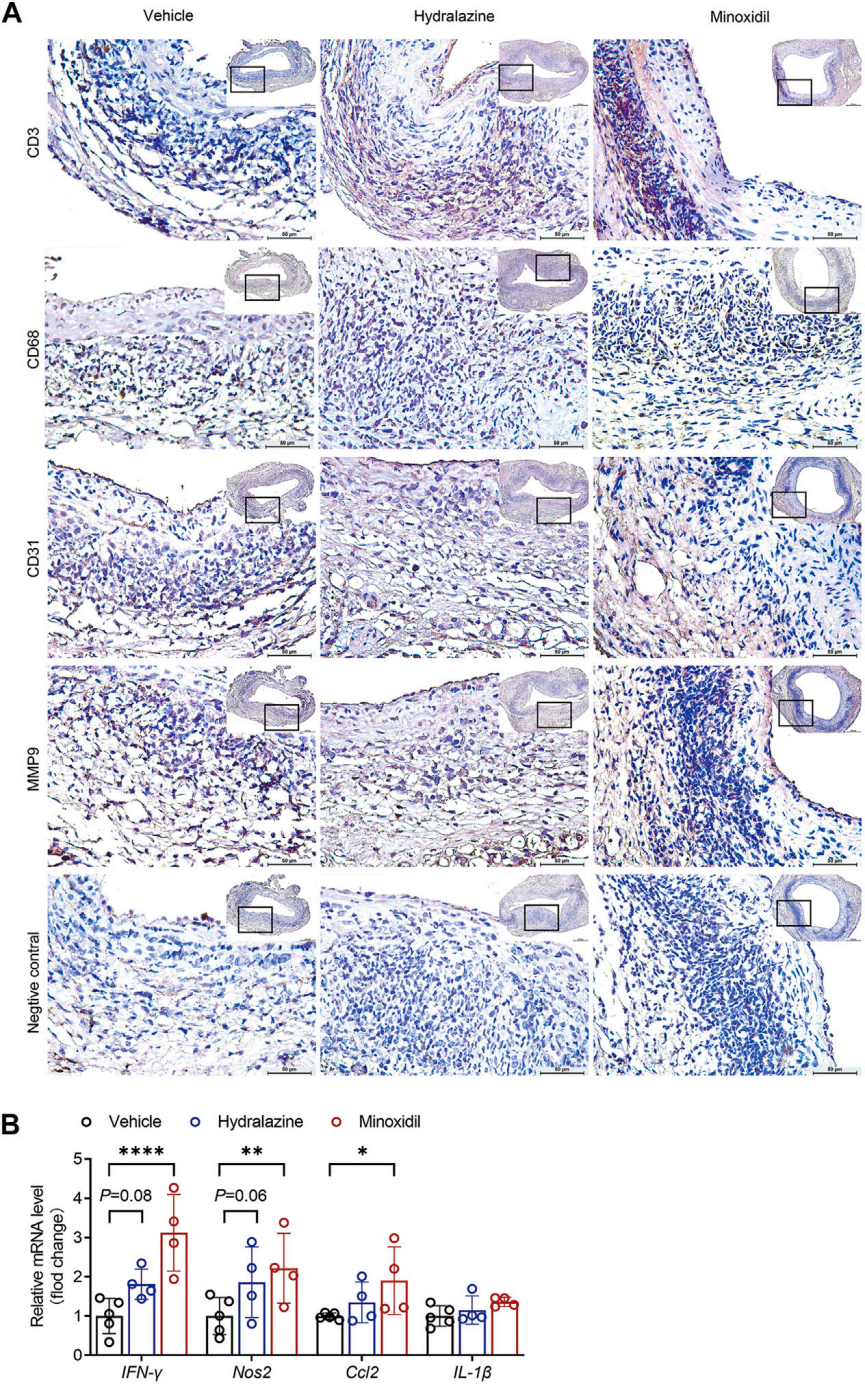
Hydrazine and minoxidil aggravated aortic inflammation in experimental AAA. **(A)** Representative immunohistological staining of CD3^+^ T cells, CD68^+^ macrophages, CD31^+^ mural angiogenesis, and MMP9. Rabbit control IgG serves as the negative control. **(B)** IFN-γ, NOS2, CCL2, and Il-1β mRNA expression were normalized to GAPDH endogenous control mRNA expression by quantitative RT-PCR. Significance was analyzed using two-way ANOVA. *p < 0.05; **p < 0.01 vs. vehicle group.

As mural inflammation promoted aortic degeneration in AAA disease, immunohistological analysis was conducted to evaluate inflammatory cell infiltration in the study. Our results revealed that both hydrazine and minoxidil aggravated mural angiogenesis and accumulation of CD3^+^ T cells, CD68^+^ macrophages, and MMP9 (Figure 4A; Supplementary Figure S2B). Furthermore, the relative mRNA level of inflammatory factors of aortic aneurysm was examined by quantitative PCR, which suggested minoxidil significantly upregulated the expression levels of *IFN-γ*, *NOS2*, and *CCL2* (Figure 4B). Similar trends were observed in the hydralazine group, but the difference was not significant, which may be due to the small sample size.

## Discussion

Antihypertensive medication is a cornerstone therapy among AAA patients with hypertension, which minimizes risks of cardiovascular events and AAA rupture. However, specific recommendations for the choice of antihypertensive medication in AAA patients remain poorly described. In the current study, we demonstrated that the PRL and PRA increased in patients with AAA compared to patients with PAD and VV, and were positively associated with AAA development after further analysis of clinical characteristics. Despite their potent blood pressure-lowering effect, direct-acting vasodilators, such as hydralazine and minoxidil, were reported to increase PRL and PRA and upregulated the renin-angiotensin system which implied an increased risk of AAA development and progression. Hence, animal experiments were performed and confirmed that administration of hydralazine and minoxidil aggravated PPE-induced experimental AAA progression with increased aortic degeneration and inflammatory infiltration. Our results highlighted the critical roles of renin in AAA disease, which raised cautionary concerns about the applications of direct vasodilators on AAA and other arterial diseases.

The involvement of the renin-angiotensin system in the pathophysiology of AAA disease has been well-established (Lu et al., 2012). Particularly secreted by juxtaglomerular cells of the kidney, renin is the rate-limiting enzyme of the renin-angiotensin system, and regulates blood pressure and cardiovascular functions. As renin catalyzes the first step in the renin-angiotensin system cascade, numerous studies have been conducted to investigate the link between PRL/PRA and the subsequent cardiovascular risk. Although the majority of these studies demonstrated a positive relationship between PRL and PRA, and cardiovascular morbidity and mortality, no definitive conclusions were obtained due to differences in the methodology among these trials (Volpe and Unger, 2013). In view of the critical role of the renin-angiotensin system in AAA disease, several previous studies demonstrated the potential role of plasma renin in AAA (Daugherty and Cassis, 2004; Steckelings and Bader, 2018). Early research addressed that excessive salt intake increased AAA incidence and rupture risk in hypertensive angiotensin and renin transgenic mice, which was protected by angiotensin converting enzyme inhibitor or angiotensin receptor blocker (Nishijo et al., 1998; Liao et al., 2001). The prorenin receptor, a specific receptor for renin and prorenin, aggravated Ang II-induced AAA on the Aope^−/−^ background, which was reversed by blocking the Ang II receptor using telmisartan (Ma et al., 2020). Furthermore, aliskiren, a direct renin inhibitor, was shown to limit atherosclerosis and AAA in an Ang II-infused mouse model by reducing prorenin receptor expression and Mitogen-activated protein kinases activity (Seto et al., 2014; Miyake et al., 2017). However, a recent case–control study reported plasma aldosterone concentrations increased in patients diagnosed with aortic dissection and aneurysms, accompanied by suppressed PRA in women rather than men (Zhu et al., 2022). In the study, we demonstrated that the PRL and PRA were increased in patients with AAA compared to those with PAD and VV, and considered risk factors for AAA, which indicated their potential role in arterial disease and AAA development. However, for patients with existing AAA, no significant correlation was found between PRL and PRA and AAA diameters. Considering the effect of Ang II on renin release through a negative feedback regulatory mechanism cannot be ignored, it suggested the roles of the renin-angiotensin system on AAA remain to be further clarified (Schweda et al., 2007).

Despite the lack of evidence that hypertension is linked to AAA progression and remains a potential risk factor for the development and prognosis of AAA (Rapsomaniki et al., 2014; Kobeissi et al., 2019; Li et al., 2019). Thus, it is encouraged to apply antihypertensive therapy in AAA patients with hypertension. Despite not being the first choice of antihypertensive agents, direct vasodilators, such as minoxidil and hydralazine, are still widely used in clinical therapeutics (Dormois et al., 1975; Mehta et al., 1975; Lu et al., 2012). Hydralazine, as a classical direct vasodilator, remains an option for the treatment of hypertension and systolic heart failure (Kandler et al., 2011). It is used as step 3 in the Hypertension Detection and Follow-up Program (HDFP) cooperative group trial and the Antihypertensive and Lipid-lowering Treatment to Prevent Heart Attack Trial (ALLHAT) to reduce the incidence of hypertension and related complications (Five-year findings of the hypertension, 1979; ALLHAT Officers and Coordinators for the ALLHAT Collaborative Research Group. The Antihypertensive and Lipid-Lowering Treatment to Prevent Heart Attack Trial, 2002). It has been proposed to inhibit IP3-induced sarcoplasmic reticulum calcium release and to inhibit myosin phosphorylation in arterial smooth muscle cells, although its mechanism is not fully elucidated (McComb et al., 2016). Minoxidil, another direct vasodilator, was introduced in the early 1970s for treating refractory hypertension in cases where multidrug regimens had failed. As an ATP-modulated potassium (K_ATP_) channel-opening agent in the vascular smooth muscle cells, minoxidil allows potassium efflux and reduces calcium influx, which ultimately relaxes vascular smooth muscle cells, dilates the aortic lumen, and lowers blood pressure (McComb et al., 2016). In our current study, we found that administration of hydralazine and minoxidil increased AAA progression with increased infiltration of local inflammatory cells and arterial degeneration. Not coincidentally, this is not the first study to investigate the relationship between vasodilating-effect drugs and AAAs. It has been reported that hydralazine showed a trend toward aggravated AAA (1.59 ± 0.21 vs. 1.38 ± 0.03; hydralazine vs. vehicle) and atherosclerotic lesion areas (3.00 ± 0.40 vs. 1.74 ± 0.50 mm^2^, hydralazine vs. vehicle, and *p* = 0.06) in Ang II-infused mice, although the differences were not significant (Cassis et al., 2009). In addition, sildenafil (Viagra), another vasodilator used to treat impotence and pulmonary hypertension, aggravated elastin degeneration and experimental AAA progression by dysregulating cyclic guanosine monophosphate and contractile signaling in vascular smooth muscle cells (Zhang et al., 2022). Other direct-acting vasodilators, such as calcium channel blockers, were also proved to increase perioperative mortality in both acute and elective aortic aneurysm surgery (Wilmink et al., 2002; Kertai et al., 2008). Consistently, as one of the dihydropyridine calcium channel blocker, amlodipine has been demonstrated to significantly promote elastin degradation and enhance matrix metalloproteinase-9 activity in experimental porcine aneurysm (Boyle et al., 1998). However, studies reported that the calcium channel blocker protected from Ang II induced AAA by reducing inflammatory infiltration and preserving eNOS coupling (Kurobe et al., 2013; Miao et al., 2015). These completely opposite results may attribute to the differences in animal models, to be precise, that Ang II negative regulates renin levels (Schweda et al., 2007). Therefore, the risk of direct-acting vasodilators to AAA diseases should be considered.

It is worth mentioning that as a direct vasodilator, minoxidil has a direct antihypertensive effect when initially administered. However, long-term administration can weaken this hypotensive effect, which is attributed to the desensitization of K^+^ channels in vascular smooth muscle cells in the aorta. This phenomenon was also observed in another ATP-dependent K^+^ channel opener, levcromakalim, which attenuated vascular relaxation in NO-donors after 2 weeks of administration in rats (Trongvanichnam et al., 1996). Now, minoxidil is widely used to treat androgenetic alopecia mainly by topical application and oral administration at a low dose (Stoehr et al., 2019; Randolph and Tosti, 2021). Adverse cardiovascular events induced by topical minoxidil suggest that it can be systemically absorbed, implying its cardiovascular risks in AAA patients (Lu et al., 2012). Although there is no clinical research exploring the relationship between minoxidil and AAA, minoxidil should be used with caution in androgenetic alopecia patients with AAA or other arterial diseases.

The risk factors for AAA are mainly old age, male gender, hypertension, and hyperlipidemia, which are consistent with the risk factors for PAD (Morley et al., 2018; Golledge, 2019). A prospective analysis of 14,148-participants also indicated that symptomatic PAD had a higher hazard ratio of incident AAA [2.96 (95% CI 1.73–5.07)], as did asymptomatic PAD [1.52 (95% CI 1.00–2.30)] (Hicks et al., 2021). Moreover, our current study showed that PRL and PRA were correlated with a higher risk of PAD [odds ratio (OR), 1.368 and 1.219, respectively] than VV (Table 3), which was consistent with previous studies. To eliminate the selective bias of the current study, we included patients with PAD and VV together in the control group and compared them separately with patients with AAAs and matched them by age, sex, and history of hypertension to enhance comparability among the clinical data. In addition, patients with PAD also showed increased PRL and PRA compared to VV disease, which raised cautionary concerns about increased renin phenotypes and applications of direct vasodilators in PAD and other arterial diseases.

Nonetheless, this study has several limitations. First, a small sample size was a major drawback of the present study. A more comprehensive analysis is needed to determine the relationship between renin and AAA disease. Second, direct-acting vasodilators are a heterogeneous group of drugs. Although hydralazine and minoxidil are the most commonly prescribed direct vasodilators, they may not be representative of all vasodilators in the study. More retrospective studies are needed to assess their safety in vascular disease. Therefore, future studies with larger samples and longitudinal measurements of people taking vasodilators are warranted to overcome the limitations to our study and establish cause-and-effect relationships.

## Conclusion

PRL and PRA are positively associated with AAA development. Hydralazine and minoxidil promote the progression of AAA in a mouse model, which might be associated with increased PRL and PRA. Direct-acting vasodilators should be used with caution in patients with AAA, as well as in those at high risk for AAA disease and other artery diseases.

## Data Availability

The original contributions presented in the study are included in the article/Supplementary Materials; further inquiries can be directed to the corresponding author.

## Supplementary material

SUPPLEMENTARY TABLE S1 Characteristics of study participants stratified by AAA occurrence.

SUPPLEMENTARY TABLE S2 Comparison of PRL and PRA between AAA and non-AAA groups.

SUPPLEMENTARY TABLE S3 Multicollinearity analysis.

SUPPLEMENTARY TABLE S4 Logistic regression analysis for the association between PRL/PRA and AAA.

SUPPLEMENTARY TABLE S5 Relationship between PRL/PRA and AAA diameter.

SUPPLEMENTARY FIGURE S1 Comparison of PRL and PRA between AAA and non-AAA groups. Plasma renin levels and renin activity were measured in the included patients to evaluate the differences in plasma renin levels and activity among the AAA (*n* = 20) and non-AAA (*n* = 40) groups. **(A)** Plasma renin level (PRL) measurement (left). Data were expressed as the median with an interquartile range. **(B)** Plasma renin activity (PRA) measurement (right). Data were expressed as the median with an interquartile range. The M-W test was applied. ***p* < 0.01 and *****p* < 0.0001.

SUPPLEMENTARY FIGURE S2 All the images of PPE-induced aneurysms and statistical analysis of immunohistological staining in the three groups. **(A)** All the images of PPE-induced aneurysms in the vehicle group (*n* = 15), hydrazine group (*n* = 9), and minoxidil group (*n* = 11) are shown. **(B)** Quantitative analysis of CD3^+^ T cells, CD68^+^ macrophages, and CD31^+^ mural angiogenesis staining in the three groups. Data are presented as the mean ± SEM. Significance was analyzed using one-way ANOVA. **p* < 0.05 vs. the vehicle group.

## Abbreviations

AAA, abdominal aortic aneurysm; PAD, peripheral artery disease; PPE, porcine pancreatic elastase; PRA, plasma renin activity; PRL, plasma renin level; VV, varicose vein.
