# Supplementary material for: Increased plasma renin by vasodilators promotes the progression of abdominal aortic aneurysm
Source: Front Pharmacol. 2023 Jun 13;14:1174278. doi: 10.3389/fphar.2023.1174278 (PMC10299739; doi:10.3389/fphar.2023.1174278)
Supplement: Supplementary file 1 [file Table1.docx]

**Supplementary Table S 1. Characteristics of Study Participants stratified by AAA Occurrence**

|  | **Non-AAA** | **AAA** | **p value** |
| --- | --- | --- | --- |
|  | **n=40** | **n=20** |  |
| **Age, y** | 67.95±6.42 | 67.6±6.34 | 0.842 |
| **Gender (women/men), n (%)** | 6/34(15/85) | 3/17(15/85) | 1.000 |
| **BMI** | 23.25±3.15 | 22.99±3.74 | 0.792 |
| **Cigarette consumption (yes), n (%)** | 22(55) | 13(65) | 0.264 |
| **CAD, n (%)** | 16(40) | 11(55） | 0.271 |
| **Diabetes, n (%)** | 14(35) | 2(10) | 0.079 |
| **Hypertnesion, n (%)** | 29(72.5) | 14(70) | 0.839 |
| **COPD, n (%)** | 4(10) | 5(25) | 0.250 |
| **CKD, n (%)** | 6(15) | 4(20） | 0.903 |
| **Duration of Hypertension, y** | 2.50(0~10.00） | 5.00(0~10.00) | 0.626 |
| **Systolic Blood Pressure, mm Hg** | 136.8±18.08 | 136.55±17.51 | 0.959 |
| **Diastolic Blood Pressure, mm Hg** | 82.25±11.15 | 78.35±15.89 | 0.274 |
| **Pulse Rate, beats per minute** | 75.43±12.43 | 75.45±14.69 | 0.995 |
| **Total Cholesterol, mmol/L** | 4.59±1 | 4.83±2.14 | 0.573 |
| **Triglyceride, mmol/L** | 1.58(1.05~2.41) | 1.59(0.98~1.99) | 0.920 |
| **LDL-C, mmol/L** | 2.95±0.74 | 3.03±1.1 | 0.751 |
| **HDL-C, mmol/L** | 1.06±0.27 | 1.05±0.26 | 0.952 |
| **ALT, U/L** | 16.25(12.45~22.60) | 14.65(9.93~18.98) | 0.201 |
| **AST, U/L** | 21.750(16.775-26.900) | 20.20(17.85~25.40) | 0.845 |
| **Serum Creatinine, μmol/L** | 73.00(63.65~88.25) | 89.60(67.95~103.08) | 0.252 |
| **Serum Uric Acid, μmol/L** | 359.85(325.25~463.43) | 437.15(306.63~514.38) | 0.480 |
| **Serum Potassium, mmol/L** | 3.98±0.47 | 3.97±0.4 | 0.952 |
| **Serum Sodium, mmol/L** | 141.24±2.86 | 141.37±3.4 | 0.882 |

Note. Values represent means ± standard deviation or count and percentage where otherwise specified. Data satisfying normal distribution were analyzed by independent sample *t* test; if not, nonparametric (M-W) and χ2 test were applied.

**Supplementary Table S2. Comparison of PRL and PRA between AAA and non-AAA Groups**

|  | **Groups** | | **U Value (M-W Test)** | **P** |
| --- | --- | --- | --- | --- |
|  | **AAA** | **Non-AAA** |  |  |
|  | **n=20** | **n=40** |  |  |
| **PRL, ulU/ml** | 17.47(11.48~65.86) | 7.02(3.67~9.65) | 72 | <0.001 |
| **PRA pg/ml** | 25.72(9.89~37.64) | 8.28(6.05~13.45) | 68 | 0.001 |

**Supplementary Table S3. Multicollinearity analysis**

|  | | **Indicators** | |
| --- | --- | --- | --- |
|  |  | **PRL** | **PRA** |
| **PRL** | **Spearman Correlation Coefficient** | 1.000 | 0.883 |
|  | **Sig.** |  | <0.001 |
|  | **Cases(n)** | 60 | 45 |
| **PRA** | **Spearman Correlation Coefficient** | 0.883 | 1.000 |
|  | **Sig.** | <0.001 |  |
|  | **Cases(n)** | 45 | 45 |

**Supplementary Table S4. Logistic Regression Analysis for the Association Between PRL/ PRA and AAA**

|  | **Unadjusted model** | | **Model 1** | | **Model 2** | |
| --- | --- | --- | --- | --- | --- | --- |
|  | **OR (95% CI)** | **P value** | **OR (95% CI)** | **P value** | **OR (95% CI)** | **P value** |
| **PRL, ulU/ml** | 1.099(1.026-1.176) | 0.007 | 1.100 (1.028-1.176) | 0.005 | 1.102(1.024-1.186) | 0.009 |

|  | **Unadjusted model** | | **Model 1** | | **Model 2** | |
| --- | --- | --- | --- | --- | --- | --- |
|  | **OR (95% CI)** | **P value** | **OR (95% CI)** | **P value** | **OR (95% CI)** | **P value** |
| **PRA, pg/ml** | 1.070(1.013-1.129) | 0.014 | 1.075 (1.015-1.138) | 0.014 | 1.088 (1.019-1.162) | 0.012 |

Note. Model 1 was adjusted for hypertension and chronic kidney disease composition ratio.

Model 2 was adjusted for hypertension, chronic kidney disease and diabetes composition ratio.

**Supplementary Table S5. The Relationship between PRL/PRA and AAA Diameter**

|  | **PRL** | | **PRA** | |
| --- | --- | --- | --- | --- |
|  | **γ** | **P value** | **γ** | **P value** |
| **Diameter (AAA+PAD)** | 0.529 | <0.001 | 0.412 | 0.021 |
| **Diameter (AAA)** | -0.025 | 0.917 | -0.335 | 0.285 |
